# Scores of peripheral neuropathic pain predicting long-term mortality in patients with type 2 diabetes: A retrospective cohort study

**DOI:** 10.3389/fendo.2022.969149

**Published:** 2022-08-16

**Authors:** Yi-Ju Liau, Shu-Fan Lin, I-Te Lee

**Affiliations:** ^1^ Department of Psychiatry, Jen-Ai Hospital, Taichung, Taiwan; ^2^ Division of Endocrinology and Metabolism, Department of Internal Medicine, Taichung Veterans General Hospital, Taichung, Taiwan; ^3^ School of Medicine, Chung Shan Medical University, Taichung, Taiwan; ^4^ School of Medicine, National Yang Ming Chiao Tung University, Taipei, Taiwan

**Keywords:** diabetes, DN4, ID pain, mortality, neuropathy, painful

## Abstract

**Objectives:**

Diabetic peripheral neuropathic pain (DPNP) is a prevalent chronic complication in patients with diabetes. Using a questionnaire is helpful for DPNP screening in outpatients. In this retrospective cohort, we aimed to examine whether DPNP diagnosed based on scoring questionnaires could predict long-term mortality in outpatients with type 2 diabetes.

**Methods:**

We enrolled 2318 patients who had joined the diabetes pay-for-performance program and completed the annual assessments, including both the identification pain questionnaire (ID pain) and Douleur Neuropathique en 4 questionnaire (DN4), between January 2013 and October 2013. Information on registered deaths was collected up to August 2019.

**Results:**

There was high consistency in the scores between the ID pain and DN4 (*r* = 0.935, P < 0.001). During the median follow-up of 6.2 years (interquartile range: 5.9−6.4 years), 312 patients deceased. Patients with an ID pain score of ≥ 2 had a higher mortality risk than those with a score of < 2 (hazard ratio [HR] = 1.394, 95%CI: 1.090−1.782), and patients with a DN4 score of ≥ 4 had a higher mortality risk than those with a score of < 4 (HR = 1.668, 95% confidence interval [CI]: 1.211−2.297). Patients consistently diagnosed with DPNP by the ID pain and DN4 had a significantly higher mortality risk (HR = 1.713, 95% CI: 1.223−2.398, P = 0.002), but not those discrepantly diagnosed with DPNP (P = 0.107), as compared with those without DPNP.

**Conclusions:**

Both the ID pain and DN4 for DPNP screening were predictive of long-term mortality in patients with type 2 diabetes. However, a discrepancy in the diagnosis of DPNP weakened the power of mortality prediction.

## Introduction

Diabetic peripheral neuropathy (DPN) is the presence of peripheral nerve dysfunction in patients with diabetes after the exclusion of other causes (1, 2). DPN is prevalent in patients with type 2 diabetes (3, 4). The prevalence of DPN was approximately 26.7% and showed high variations in ethnicity based on interviews with patients with type 2 diabetes across 14 countries in the International Prevalence and Treatment of Diabetes and Depression study (5). In Taiwan, the prevalence of DPN was 26.8% according to the Neurological Symptom Score questionnaire (6), and was 34.5% by physical examination according to the Michigan Neuropathy Screening Instrument (MNSI) criteria (7). DPN has become a heavy burden on health and the economy due to subsequent foot complications or pain relief treatments (8, 9). An investigation determined that most patients with diabetes did not undergo foot examination in the rural community (7). Non-invasive questionnaires would be helpful for early screening of DPN in clinical practice (6).

DPN is a heterogeneous group of disorders with diverse clinical manifestations (1). More than 40% of patients with DPN suffer from peripheral neuropathic pain (10). Diabetic peripheral neuropathic pain (DPNP) is a common problem and patients with DPNP should be treated for symptom relief (10, 11). The presence of pain is associated with several comorbidities, including mental disorders, and has been reported to increase health-care costs (9–12). Since objective measurements are not sensitive for evaluating painful symptoms, the diagnosis of DPNP is challenging. To facilitate awareness of neuropathic pain, several questionnaires have been developed. The identification pain questionnaire (ID pain) is a self-administered questionnaire with the six items (13). The Douleur Neuropathique en 4 questionnaire (DN4) is a clinician-administered questionnaire with ten items (14). The DN4 has been validated in the assessment of DPNP with a high diagnostic accuracy (15), and there is good consistency between DN4 and ID pain in the diagnosis of neuropathic pain (16).

It was reported that DPN diagnosed based on low signal amplitude and slow conduction velocity in a nerve conduction study is associated with total mortality in patients with type 2 diabetes (17). DPN diagnosed based on the MNSI could predict a high incidence of cardiovascular (CV) disease; however, it was not significantly associated with total mortality in patients with type 2 diabetes (18). Notably, DPNP based on electronic health records might be associated with a significantly higher mortality risk than DPN without pain in patients with type 2 diabetes (19). We hypothesized that pain symptoms are predictive of mortality in patients with type 2 diabetes. Therefore, we aimed to examine the association between mortality and neuropathic pain screened using the ID pain and DN4 in patients with type 2 diabetes.

## Materials and methods

### Study design and participants

This retrospective cohort study was conducted at Taichung Veterans General Hospital. The inclusion criteria were [1] adult patients who had joined the diabetes pay-for-performance program and [2] patients who had completed both the ID pain and DN4 for DPNP screening between January 2013 and October 2013. The exclusion criteria were [1] end-stage renal disease; [2] an open wound or amputation of the lower extremities; [3] diabetes other than type 2; [4] malignancy, psychiatric disorders, current use of any pain medication involving the central nervous system, other severe systemic diseases, or pregnancy; and [5] other conditions unsuitable for the study.

### Procedure

The diabetes pay-for-performance program is an important health policy for clinical diabetic care (20). Patients who had been diagnosed with diabetes and repeatedly visited our hospital within ninety days were encouraged to participate in the program (21). The completion of a comprehensive survey was requested in this program, including diabetic neuropathy screening every year. The questionnaires for scoring DPNP were administrated by a well-trained educator at our Diabetes Care Center. We only collected the last assessment records for the participants who had undergone repeated annual comprehensive assessments in the enrollment period.

The six items of ID pain were reported by patients themselves: each item was scored 0 when a participant answered “no”. Items 1−5 received a score of 1 when a participant answered “yes”, and Item 6 received a score of -1 when a participant answered “yes” (13). The total ID pain score ranges from -1 to 5, and a score ≥ 2 indicates that a diagnosis of peripheral neuropathic pain is likely (22). The Mandarin Chinese version of the ID pain has been validated in previous studies (22–24). The ten items of DN4 were assessed by a trained educator certified by the Taiwanese Association of Diabetes Educators: each item was assigned a score of 0 when a participant answered “no” and a score of 1 when a participant answered “yes”. The total DN4 score ranges from 0 to 10, and a score ≥ 4 indicates a diagnosis of peripheral neuropathic pain (14, 15). The Mandarin Chinese version of the DN4 has been validated previously (25).

Furthermore, laboratory data, including plasma glucose, glycated hemoglobin (HbA1c), serum levels of total cholesterol, high-density lipoprotein (HDL) cholesterol, triglycerides, and creatinine, and urinary levels of albumin and creatinine, were collected during the annual comprehensive survey. Information on the use of antidiabetic drugs, antihypertensive drugs, antiplatelet drugs, and statins was also collected.

### Laboratory measurements

Plasma glucose levels were determined using the oxidase-peroxidase method (Wako Diagnostics, Tokyo, Japan). HbA1c levels were determined by cation-exchange high performance liquid chromatography (National Glycohemoglobin Standardization Program certified; G8, TOSOH, Tokyo, Japan). Serum concentrations of total cholesterol, HDL cholesterol, and triglycerides were determined using enzymatic methods (Advia 1800, Siemens, New York, USA). Creatinine levels were determined using the Jaffé method (Advia 1800, Siemens, New York, USA). Urinary albumin was determined using the polyethylene glycol enhanced immunoturbidimetric method (Advia 1800, Siemens, New York, USA). The estimated glomerular filtration rate (eGFR) was calculated according to the Chronic Kidney Disease Epidemiology Collaboration (CKD-EPI) equation as follows: 141 × (serum creatinine [mg/dL]/0.9)^-0.411^ × 0.993^age (year)^ in men with serum creatinine ≤ 0.9 mg/dL; 141 × (serum creatinine [mg/dL]/0.9)^-1.209^ × 0.993^age (year)^ in men with serum creatinine > 0.9 mg/dL; 144 × (serum creatinine [mg/dL]/0.7)^-0.329^ × 0.993^age (year)^ in women with serum creatinine ≤ 0.7 mg/dL; or 144 × (serum creatinine [mg/dL]/0.9)^-1.209^ × 0.993^age (year)^ in women with serum creatinine > 0.7 mg/dL (26, 27). CKD was defined as an eGFR < 60 mL/min/1.73 m^2^ (2, 28). The urinary albumin-to-creatinine ratio (UACR) was calculated as follows: albumin (mg)/creatinine (g), and albuminuria was defined as a UACR ≥ 300 mg/g (2, 28). Hypertension was defined as a systolic blood pressure ≥ 140 mmHg, a diastolic blood pressure ≥ 90 mmHg, or current use of an antihypertensive drug. Hypertriglyceridemia was defined as serum triglycerides ≥ 150 mg/dL (1.7 mmol/L), and low HDL cholesterol was defined as a serum HDL cholesterol < 40 mg/dL (1.0 mmol/L) in men or < 50 mg/dL (1.3 mmol/L) in women (29).

### Statistical analysis

We present the mean ± standard deviation (SD) for continuous variables and numbers with percentages (%) for categorical data. The clinical variables were tested for statistically significant differences using Student’s t test for continuous variables between two groups, and the χ2 test for categorical variables. The relationship between the scores of the questionnaires was determined by Pearson’s correlation.

The reproducibility of the ID pain and DN4 was examined in a group of 51 subjects by repeated assessments on different days. The median period between repeated measurements was 161 days (interquartile range [IQR] between 112 and 252 days). Highly positive correlations of ID pain score (correlation coefficient [*r*] = 0.870, P < 0.001) and DN4 score (*r* = 0.789, P < 0.001) were observed between the first and second measurements. The 95% confidence intervals (CIs) were 0.078 ± 0.158 for the bias of the ID pain and 0.020 ± 0.221 for the DN4 between repeated measurements based on Bland-Altman plots (30).

The primary endpoint was the occurrence of all-cause mortality. Information on deaths registered through August 31, 2019, was obtained from the Ministry of Health and Welfare, Executive Yuan, Taiwan. The univariate cumulative risk for all-cause mortality was assessed by Kaplan-Meier analysis, and significance was tested by the log-rank test. The receiver operating characteristic (ROC) curve was used to determine the prediction for mortality in patients with DPNP according to different criteria. Multivariate Cox proportional hazards regression analyses were used to determine the primary endpoint according to the diagnosis of DPNP. The CV mortality was defined based on the International Classification of Diseases 10th Revision, and the codes included those for heart diseases (I01−I25, I27, and I30−I52), stroke (I60−I69), and peripheral artery disease (I70.2−I75). A two-tailed P value of < 0.05 was considered statistically significant. Statistical analysis was performed using SPSS 22.0 (IBM, Armonk, NY, USA).

## Results

Of a total of 2318 patients, 505 (21.8%) and 179 (7.7%) were diagnosed with DPNP based on ID pain scores ≥ 2 and DN4 score ≥ 4, respectively. The baseline characteristics of the patients grouped by DPNP are shown in **Table 1**. The patients with DPNP were significantly older than those without DPNP (P < 0.001 based on either the ID pain or DN4). The proportions of males were significantly lower among patients with DPNP than among those without DPNP (P < 0.001 based on the ID pain and P = 0.004 based on the DN4, respectively). The patients with DPNP had a significantly longer diabetes duration than those without DPNP (P < 0.001 based on either the ID pain or DN4). There were significantly higher proportions of previous coronary artery disease (CAD) history and hypertension in the patients with DPNP than in those without DPNP (P < 0.05 based on either the ID pain or DN4). The patients with DPNP had significantly higher HbA1c levels than those without DPNP (P = 0.013 based on the ID pain and P = 0.007 based on the DN4, respectively). The patients with DPNP had significantly lower eGFR and higher UACR than those without DPNP (P < 0.001 based on either the ID pain or DN4). There were significantly higher proportions of antiplatelet agent, antihypertensive drug, and insulin injection therapy use in the patients with DPNP than in those without DPNP (P < 0.01 based on either the ID pain or DN4). Among the oral antihyperglycemic drugs, only the proportions of metformin use were significantly different between the patients with and without DPNP (P = 0.009 based on the ID pain and P = 0.041 based on the DN4, respectively).

**Table 1:** The baseline characteristics of all patients, categorized by peripheral neuropathic pain scores.

	All (n = 2318)		ID pain score < 2(n = 1813)		ID pain score ≥ 2(n = 505)		P	DN4 score < 4 (n = 2139)		DN4 score ≥ 4 (n = 179)		P
Age (year)	63.1	± 13.2	62.4	± 13.1	65.6	± 12.9	<0.001	62.6	± 13.1	68.7	± 13.2	<0.001
Male, n (%)	1307	(56.4%)	1079	(59.5%)	228	(45.1%)	<0.001	1225	(57.3%)	82	(45.8%)	0.004
Diabetes duration (year)	10.1	± 8.2	9.6	± 8.0	12.0	± 8.8	<0.001	9.9	± 8.1	12.7	± 9.2	<0.001
Currently smoking, n (%)	236	(10.2%)	184	(10.1%)	52	(10.3%)	0.989	221	(10.3%)	15	(8.4%)	0.483
CAD history, n (%)	699	(30.2%)	518	(28.6%)	181	(35.8%)	0.002	632	(29.5%)	67	(37.4%)	0.034
Hypertension, n (%)	1776	(76.6%)	1364	(75.2%)	412	(81.6%)	0.003	1622	(75.8%)	154	(86.0%)	0.003
BMI (kg/m^2^)	25.7	± 4.1	25.7	± 4.0	25.9	± 4.4	0.231	25.7	± 4.0	25.9	± 4.7	0.565
Systolic BP (mmHg)	130.9	± 13.6	130.9	± 13.5	131.2	± 14.0	0.615	130.9	± 13.4	131.7	± 15.7	0.443
Diastolic BP (mmHg)	77.5	± 8.9	77.4	± 8.8	77.6	± 9.2	0.634	77.5	± 8.7	77.8	± 10.3	0.638
ID pain score	0.7	± 1.2	0.2	± 0.4	2.7	± 0.8	<0.001	0.5	± 0.9	3.2	± 0.9	<0.001
DN4 score	0.9	± 1.4	0.3	± 0.6	3.2	± 1.1	<0.001	0.6	± 1.0	4.5	± 0.7	<0.001
Fasting glucose (mmol/L)	8.7	± 3.6	8.6	± 3.5	8.9	± 3.9	0.137	8.7	± 3.6	9.0	± 3.9	0.190
HbA1c (%)	7.8	± 1.7	7.8	± 1.7	8.0	± 1.8	0.013	7.8	± 1.7	8.2	± 1.8	0.007
Total cholesterol (mmol/L)	4.4	± 1.0	4.4	± 1.0	4.4	± 1.0	0.953	4.4	± 1.0	4.4	± 0.9	0.578
HDL cholesterol (mmol/L)	1.3	± 0.4	1.3	± 0.4	1.3	± 0.4	0.594	1.3	± 0.4	1.3	± 0.4	0.156
Triglycerides (mmol/L)	1.7	± 1.7	1.7	± 1.7	1.7	± 1.6	0.590	1.7	± 1.6	1.7	± 2.2	0.876
eGFR (mL/min/1.73 m^2^)	77.9	± 25.2	79.4	± 24.7	72.5	± 26.1	<0.001	78.7	± 24.8	67.8	± 26.9	<0.001
UACR	173.0	± 521.5	142.3	± 456.0	283.2	± 698.0	<0.001	154.3	± 483.8	395.9	± 821.2	<0.001
Antiplatelet drugs, n (%)	653	(28.2%)	476	(26.3%)	177	(35.0%)	<0.001	587	(27.4%)	66	(36.9%)	0.009
Statins, n (%)	1502	(64.8%)	1170	(64.5%)	332	(65.7%)	0.653	1386	(64.8%)	116	(64.8%)	0.999
Antihypertensive drugs, n (%)	1480	(63.8%)	1116	(61.6%)	364	(72.1%)	<0.001	1345	(62.9%)	135	(75.4%)	0.001
ACE inhibitors or ARBs, n (%)	1244	(53.7%)	927	(51.1%)	317	(62.8%)	<0.001	1129	(52.8%)	115	(64.2%)	0.004
α-Blockers, n (%)	253	(10.9%)	191	(10.5%)	62	(12.3%)	0.303	228	(10.7%)	25	(14.0%)	0.216
β-Blockers, n (%)	494	(21.3%)	377	(20.8%)	117	(23.2%)	0.275	453	(21.2%)	41	(22.9%)	0.655
CCBs, n (%)	140	(6.0%)	99	(5.5%)	41	(8.1%)	0.035	126	(5.9%)	14	(7.8%)	0.380
Diuretics, n (%)	344	(14.8%)	235	(13.0%)	109	(21.6%)	<0.001	293	(13.7%)	51	(28.5%)	<0.001
Insulin therapy, n (%)	607	(26.2%)	421	(23.2%)	186	(36.8%)	<0.001	537	(25.1%)	70	(39.1%)	<0.001
Oral antihyperglycemic drugs	2074	(89.5%)	1624	(89.6%)	450	(89.1%)	0.826	1914	(89.5%)	160	(89.4%)	0.999
Insulin secretagogues, n (%)	1194	(51.5%)	937	(51.7%)	257	(50.9%)	0.792	1096	(51.2%)	98	(54.7%)	0.410
Metformin, n (%)	1251	(54.0%)	1005	(55.4%)	246	(48.7%)	0.009	1168	(54.6%)	83	(46.4%)	0.041
Thiazolidinediones, n (%)	494	(21.3%)	383	(21.1%)	111	(22.0%)	0.724	449	(21.0%)	45	(25.1%)	0.227
DPP4 inhibitors, n (%)	1063	(45.9%)	824	(45.4%)	239	(47.3%)	0.485	970	(45.3%)	93	(52.0%)	0.104
Mortality, n (%)	312	(13.5%)	213	(11.7%)	99	(19.6%)	<0.001	266	(12.4%)	46	(25.7%)	<0.001
Incidence of mortality*	2.3		2.0		3.5		<0.001	2.1		4.8		<0.001
CV mortality, n (%)	60	(2.6%)	41	(2.3%)	19	(3.8%)	0.085	51	(2.4%)	9	(5.0%)	0.058
Incidence of CV mortality*	0.4		0.4		0.7		0.036	0.4		0.9		0.013

Continuous data are expressed as the mean ± standard deviation. Categorical data are expressed as numbers (percentages).

*Unit: deaths/100 person-years. Statistical significance was detected using the log rank test.

ACE, angiotensin-converting enzyme; ARBs, angiotensin receptor blockers; BMI, body mass index; BP, blood pressure; CAD, coronary artery disease; CCBs, calcium channel blockers; CV, cardiovascular; DN4, Douleur Neuropathique en 4 questionnaire; DPP4, dipeptidyl peptidase-4; eGFR, estimated glomerular filtration rate; HbA1c, hemoglobin A1c; HDL, high-density lipoprotein; ID pain, identification pain questionnaire; UACR, urine albumin-creatinine ratio.

**Figure 1:**
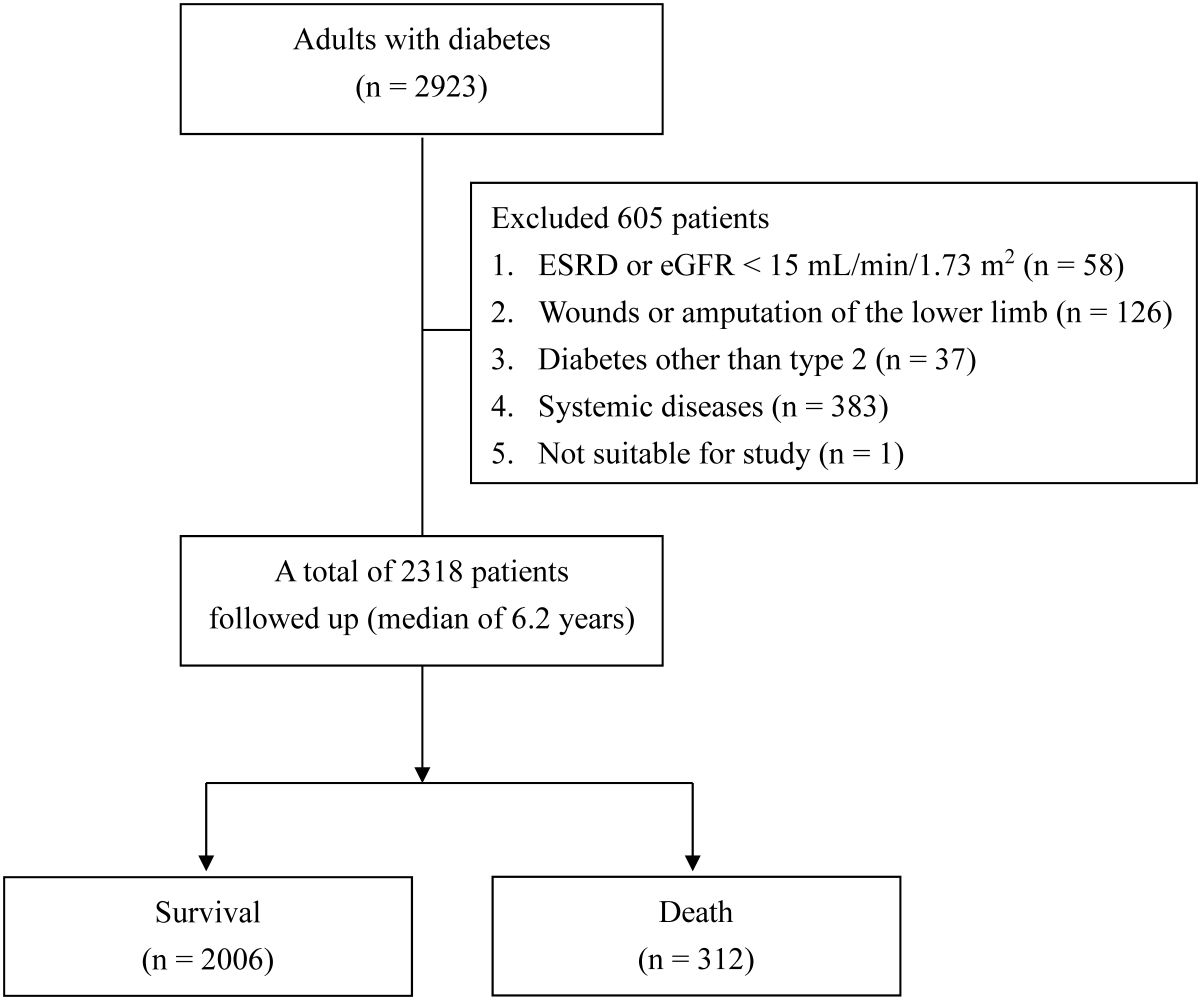
Flow diagram of the enrollment process for study subjects.

During a median follow-up of 6.2 years (IQR: 5.9−6.4 years), 312 patients died (**Figure 1**). The incidences of mortality were 3.5 deaths/100 person-years in patients with ID pain scores ≥ 2 and 4.8 deaths/100 person-years in patients with DN4 scores ≥ 4; and 2.0 deaths/100 person-years in patients with ID pain scores < 2 and 2.1 deaths/100 person-years in patients with DN4 scores < 4. Notably, 60 of 312 deaths (19.2%) caused by CV diseases (**Table 1**). The CV−total mortality ratios were not significantly different between patients with ID pain scores ≥ 2 and < 2 (19.2% vs. 19.2%, P = 0.999). Similarly, the CV−total mortality ratios were not significantly different between patients with DN4 scores ≥ 4 and < 4 (19.6% vs. 19.2%, P = 0.999).

**Figure 2:**
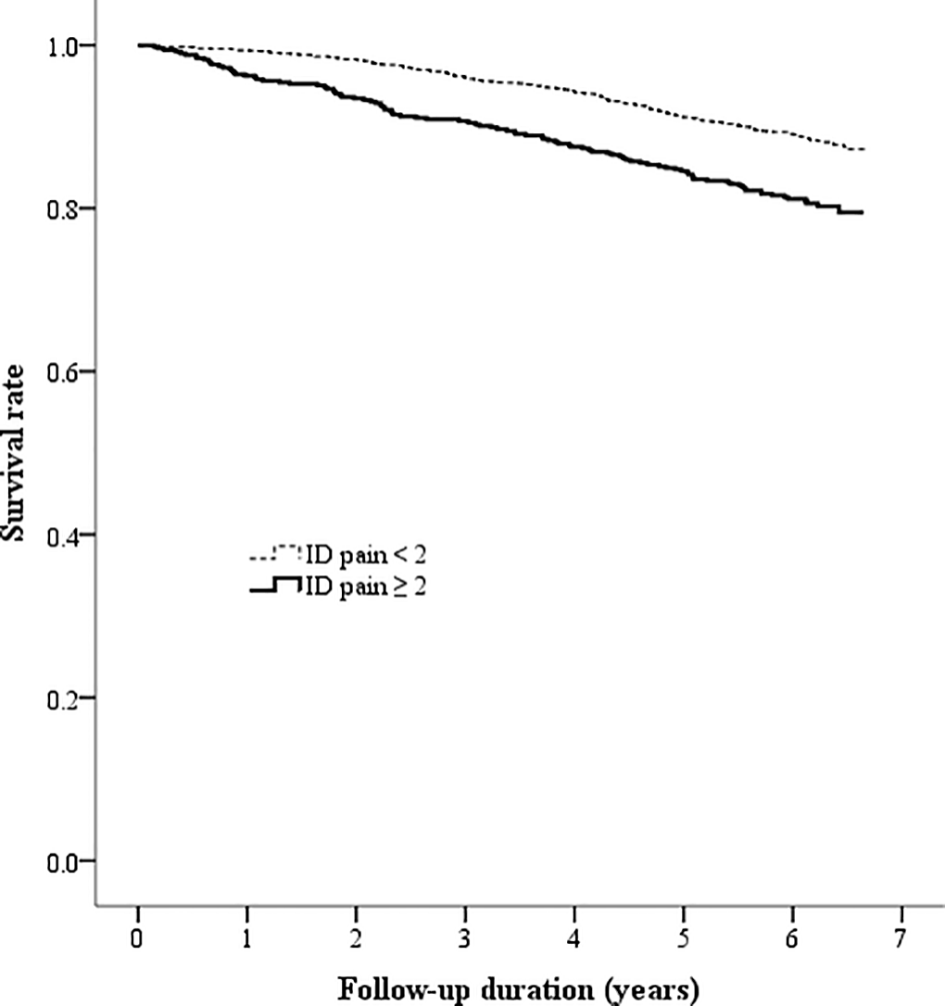
Kaplan-Meier curves showing the survival rate between the two patient groups categorized by the baseline ID pain score (ID pain = identification pain questionnaire).

**Figure 3:**
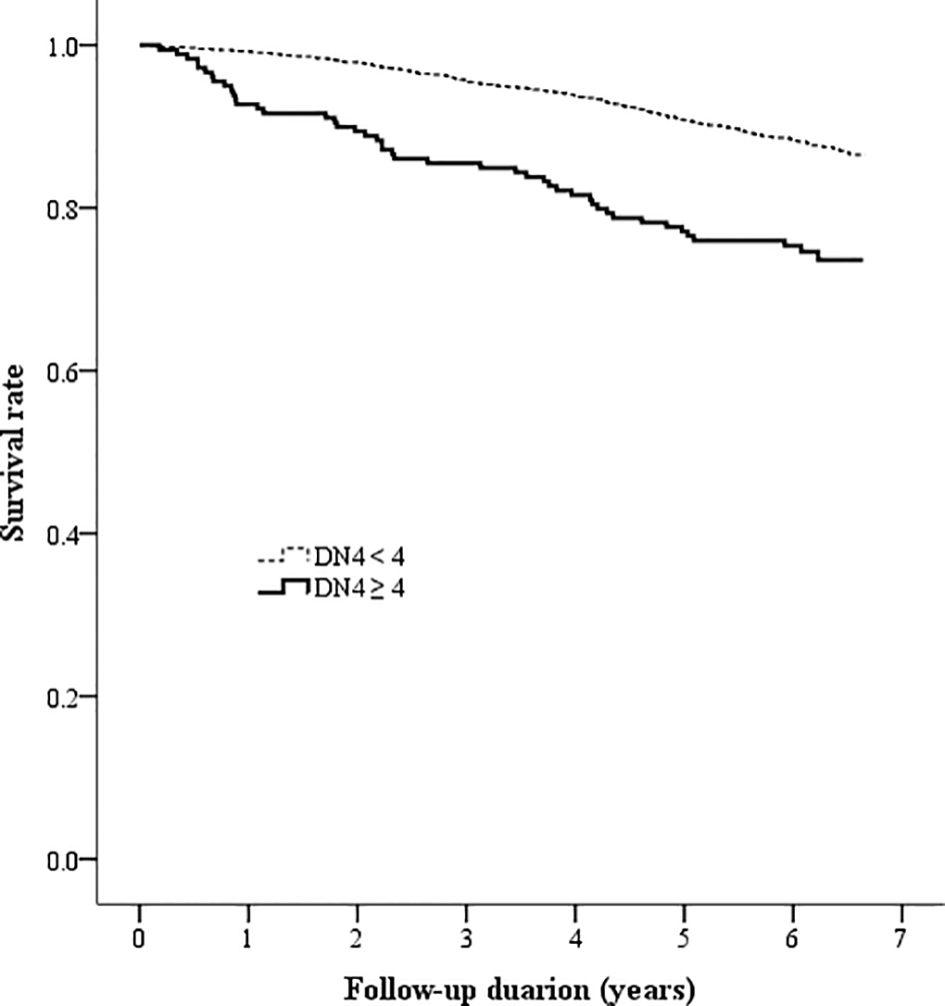
Kaplan-Meier curves showing the survival rate between the two patient groups categorized by the baseline DN4 score (DN4 = Douleur Neuropathique en 4 questionnaire).

The Kaplan-Meier analysis showed that the survival rates were significantly different between patients with and without DPNP (log-rank test P < 0.001 based on the ID pain, **Figure 2**; or based on the DN4, **Figure 3**). According to each item in the ID pain, all positive symptoms were significantly predictive of mortality, except the Item 6, which was designed to exclude DPNP; according to each item in the DN4, all positive symptoms were significantly predictive of mortality, except the Items 2 and 4, as few patients experienced the symptoms (**Table 2**). Using multivariable Cox proportional hazard regression models, the ID pain score was a significant predictor for total mortality with an HR of 1.394 (95% CI: 1.090−1.782, P = 0.008; **Table 3**), and the DN4 was also a significant predictor for total mortality with an HR of 1.668 (95% CI: 1.211−2.297, P = 0.002; **Table 3**) after adjusting for age, sex, hypertension, CAD history, diabetes duration, HbA1c level, HDL cholesterol level, CKD, the UACR, and the use of antiplatelet drugs, antihypertensive drugs, insulin, and metformin.

**Table 2:** The number (percentage) and mortality HR (95% CI) of patients with positive symptoms for each question item.

	Pain description	Total(n= 2318)		Survival(n = 2006)		Death(n = 312)		P	HR	95% CI		P
ID pain score ≥ 2		505	(21.8%)	406	(20.2%)	99	(31.7%)	<0.001	1.777	(1.400,	2.255)	<0.001
ID pain Q1	Pins and needles	326	(14.1%)	260	(13.0%)	66	(21.2%)	<0.001	1.757	(1.339,	2.306)	<0.001
ID pain Q2	Hot or burning	159	(6.9%)	130	(6.5%)	29	(9.3%)	0.087	1.485	(1.013,	2.176)	0.042
ID pain Q3	Numbness	817	(35.2%)	682	(34.0%)	135	(43.3%)	0.002	1.456	(1.164,	1.822)	<0.001
ID pain Q4	Electric shocks	293	(12.6%)	233	(11.6%)	60	(19.2%)	<0.001	1.743	(1.315,	2.309)	<0.001
ID pain Q5	Worse with the touch of clothing or bed sheets	100	(4.3%)	78	(3.9%)	22	(7.1%)	0.016	1.810	(1.174,	2.793)	0.007
ID pain Q6*	Limited to joints	8	(0.3%)	8	(0.4%)	0	(0.0%)	0.608	-^†^			
DN4 score ≥ 4		179	(7.7%)	133	(6.6%)	46	(14.7%)	<0.001	2.344	(1.714,	3.205)	<0.001
DN4 Q1	Burning	159	(6.9%)	130	(6.5%)	29	(9.3%)	0.087	1.486	(1.014,	2.178)	0.042
DN4 Q2*	Painful cold	37	(1.6%)	29	(1.4%)	8	(2.6%)	0.145	1.692	(0.838,	3.414)	0.142
DN4 Q3	Electric shocks	295	(12.7%)	235	(11.7%)	60	(19.2%)	<0.001	1.728	(1.304,	2.290)	<0.001
DN4 Q4*	Tingling	18	(0.8%)	16	(0.8%)	2	(0.6%)	0.999	0.878	(0.219,	3.528)	0.855
DN4 Q5	Pins and needles	307	(13.2%)	243	(12.1%)	64	(20.5%)	<0.001	1.811	(1.376,	2.384)	<0.001
DN4 Q6	Numbness	816	(35.2%)	681	(33.9%)	135	(43.3%)	0.002	1.459	(1.166,	1.826)	<0.001
DN4 Q7	Itching	150	(6.5%)	119	(5.9%)	31	(9.9%)	0.011	1.735	(1.197,	2.515)	0.004
DN4 Q8	Hypoesthesia to touch	44	(1.9%)	33	(1.6%)	11	(3.5%)	0.041	2.000	(1.095,	3.653)	0.024
DN4 Q9	Hypoesthesia to pinprick	264	(11.4%)	180	(9.0%)	84	(26.9%)	<0.001	3.213	(2.501,	4.126)	<0.001
DN4 Q10	Brushing	90	(3.9%)	66	(3.3%)	24	(7.7%)	<0.001	2.261	(1.491,	3.428)	<0.001

*P value detected using Fisher’s test. ^†^There was no mortality in the patients with a positive answer to ID pain Q6.

CI, confidence interval; DN4, Douleur Neuropathique en 4 questionnaire; HR, hazard ratio for mortality using univariable Cox regression model; ID pain, identification pain questionnaire.

**Table 3:** Cox proportional hazard regression models for the association between DPNP and mortality.

	Model 1				Model 2				Model 3			
	HR	95%	CI	P	HR	95%	CI	P	HR	95%	CI	P
Screening by ID pain score												
ID pain score < 2 (reference)	1.000				1.000				1.000			
DPNP based on ID pain score ≥ 2	1.684	(1.324,	2.143)	<0.001	1.490	(1.167,	1.902)	0.001	1.394	(1.090,	1.782)	0.008
Screening by DN4 score												
DN4 score < 4 (reference)	1.000				1.000				1.000			
DPNP based on DN4 score ≥ 4	1.996	(1.457,	2.736)	<0.001	1.692	(1.230,	2.329)	0.001	1.668	(1.211,	2.297)	0.002
Screening by ID pain and DN4 scores												
No neuropathic pain	1.000				1.000				1.000			
DPNP by either the ID pain or DN4	1.511	(1.131,	2.020)	0.005	1.365	(1.018,	1.829)	0.038	1.273	(0.949,	1.709)	0.107
DPNP by both ID pain and DN4	2.122	(1.524,	2.954)	<0.001	1.787	(1.278,	2.498)	<0.001	1.713	(1.223,	2.398)	0.002

Model 1: adjusted for age and sex.

Model 2: adjusted for age, sex, hypertension, CAD history, diabetes duration, HbA1c, HDL cholesterol, CKD, and UACR.

Model 3: adjusted for age, sex, hypertension, CAD history, diabetes duration, HbA1c, HDL cholesterol, CKD, UACR, and uses of antiplatelet drugs, antihypertensive drugs, insulin, and metformin.

CAD, coronary artery disease; CKD, chronic kidney disease; CI, confidence interval; DN4, Douleur Neuropathique en 4 questionnaire; DPNP, diabetic peripheral neuropathic pain; HbA1c, hemoglobin A1c; HDL, high-density lipoprotein; HR, hazard ratio; ID pain, identification pain questionnaire; UACR, urine albumin-creatinine ratio.

**Figure 4:**
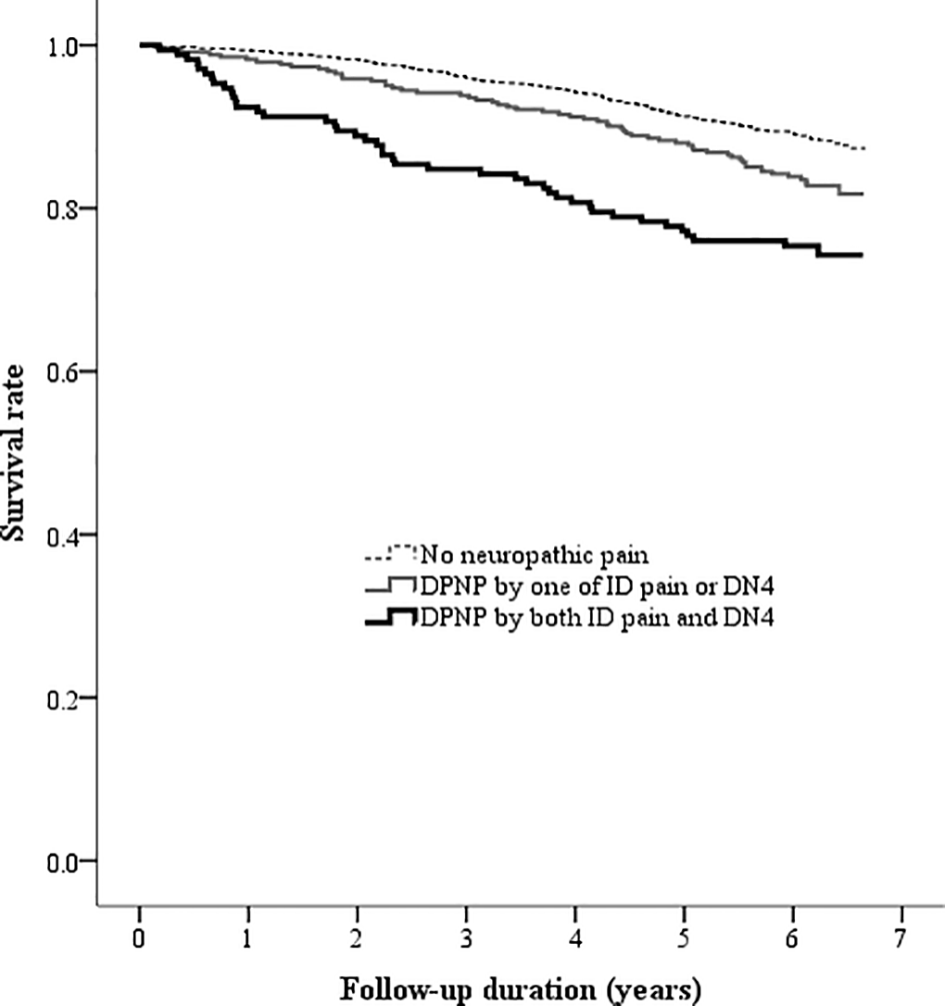
Kaplan-Meier curves showing the survival rate among the three groups categorized by the DPNP diagnosis. (DN4, Douleur Neuropathique en 4 questionnaire; DPNP, diabetic peripheral neuropathic pain; ID pain, identification pain questionnaire).

There was a high correlation between the ID pain and DN4 scores (*r* = 0.935, P < 0.001). According to the criteria of peripheral neuropathic pain based on these two scoring questionnaires, 1805 (77.9%) patients were consistently grouped as no DPNP, and 171 (7.4%) patients were consistently grouped as DPNP. However, 8 (0.3%) patients were grouped as no DPNP based on the ID pain score but were discrepantly grouped as DPNP based on the DN4 score; 334 (14.4%) patients were grouped as DPNP based on the ID pain score but were discrepantly as no DPNP based on the DN4 score. The consistent group of diagnosed DPNP showed a sensitivity of 17.0%, a specificity of 92.6%, a positive predictive value (PPV) of 25.1%, and a negative predictive value (NPV) of 88.4% to predict mortality; the discrepant group of DPNP diagnoses showed a sensitivity of 21.9%, a specificity of 84.9%, a PPV of 17.3%, and a NPV of 88.4% to predict mortality during follow-up (**Table 4**). The consistent group of diagnosed DPNP based on the ID pain and DN4 had the highest mortality risk (**Figure 4**), and was significantly predictive of mortality compared to the consistent group of no DPNP (HR = 1.713, 95% CI: 1.223−2.398, P =0.002; **Table 3**). However, the discrepant group of DPNP diagnoses weakened the mortality prediction toward a null hypothesis (HR = 1.273, 95% CI: 0.949−1.709; P = 0.107) after adjusting for the associated factors.

**Table 4:** Prediction of mortality by DPNP using different questionnaire criteria.

	ROC curve analysis		Sensitivity	Specificity	PPV	NPV
	AUC (95%CI)	P				
DPNP by both ID pain and DN4	0.584 (0.508, 0.588)	0.014	17.0%	92.6%	25.1%	88.4%
DPNP by either ID pain or DN4 alone	0.534 (0.496, 0.572)	0.068	21.9%	84.9%	17.3%	88.4%

AUC, area under the curve; DN4, Douleur Neuropathique en 4 questionnaire; DPNP, diabetic peripheral neuropathic pain; ID pain, identification pain questionnaire; NPV, negative predictive value; PPV, positive predictive value; ROC, receiver operating characteristic.

## Discussion

The main finding of our study revealed that the scores of peripheral neuropathic pain using the ID pain and DN4 were predictive of total mortality in patients with type 2 diabetes, with a median follow-up duration of 6.2 years. There was a high consistency in the diagnosis of DPNP between the ID pain and DN4 scores. Notably, a discrepancy in the diagnosis of DPNP attenuated the prediction of long-term mortality. The ID pain and DN4 are widely used tools for screening neuropathic pain, and Padua et al. (16) reported a low discrepancy rate of 16% between the ID pain and DN4 in diagnosing neuropathic pain in Italians with established peripheral nerve diseases. A strength of the present study is that use of these two questionnaires to actively screen peripheral neuropathic pain could predict total mortality risk in patients with type 2 diabetes. In line with our findings, Lapin et al. (19) reported that peripheral neuropathic pain defined by medical records or medication usage predicted CV disease and total mortality in patients with type 2 diabetes. Since chronic pain has been reported as a CV risk factor (31), the early detection of peripheral neuropathic pain is important to evaluate the associated comorbidities and to categorize the long-term mortality risk for patients with type 2 diabetes (32).

The mechanisms of pain are complex in diabetes. Generally, peripheral neuropathic pain might result from the facilitated excitation of pain signals and reduced inhibition of the central nervous system (33). Small-fiber neuropathy can generate action potentials even without an external stimulus in patients with diabetes (34). Several CV risk factors, including metabolic disorders and vasculopathy, are associated with neural injury (35). In the present study, higher pain scores were associated with age, diabetes duration, hypertension, CAD, hyperglycemia, CKD, and albuminuria. DPNP was an independent predictor of mortality after adjusting for all assessed potential risk factors; however, several nontraditional CV risks associated with DPN were not assessed in the present study (36). Variability of fasting glucose was reported to be associated with DPNP, peripheral artery disease, and mortality (37–39). Retinopathy was reported to be associated with DPN, CKD, and mortality (40, 41). Endothelial dysfunction, a well-known CV risk factor, was also reported to be associated with DPN (42).

In addition to comorbidities between DPNP and CV disease, imbalanced glycolytic metabolites and decreased neuroprotective factors may be involved in the mechanism between DPNP and mortality. Methylglyoxal, which might be generated during glycolysis in cells, is associated with the hyperexcitability of neurons (43). In patients with type 2 diabetes, the plasma methylglyoxal level was higher in individuals with DPNP than in those without pain (44). Hannssen et al. (45) reported that a high plasma methylglyoxal level was associated with an increased risk of diabetic nephropathy, CV events, and all-cause mortality. Alterations in the function and structure of the brain were observed in patients with DPN. N-acetyl aspartate was decreased in the thalamus of patients with DPNP (46), and reduced inhibition of central nervous system might be associated with psychological comorbidities (47–49). A decrease in serum brain-derived neurotrophic factor (BDNF), a neurotrophin family member, is associated with peripheral neuropathy in patients with type 2 diabetes (50). Previously, we reported that a low serum BDNF level was also associated with depressive symptoms and predictive of long-term mortality (51, 52).

The prevalence of peripheral neuropathic pain was approximately 13% in patients with diabetes (53, 54), and was higher in patients with type 2 diabetes than in those with type 1 diabetes (54, 55). According to electronic health records, approximately 20.7% of the patients with type 2 diabetes had experienced any peripheral neuropathic pain (19). An optimal cutoff ID pain score of ≥ 2 with a sensitivity of 77% is suggested for the diagnosis of peripheral neuropathic pain in the Taiwanese population (24). In the present study, the prevalence of DPNP was 21.8% based on the cutoff ID pain score of ≥ 2. Notably, a low prevalence (7.7%) of DPNP was found using the cutoff DN4 score of ≥ 4 in the present study. It has been reported that an optimal cutoff DN4 score of ≥ 3 with a similar sensitivity of 77% is suggested for the diagnosis of peripheral neuropathic pain in the Taiwanese population (25). The prevalence of DPNP was 15.7% when using the criterion of a cutoff DN4 score ≥ 3 (data not shown) in the present study, and was similar to the prevalence of 17.9% in outpatients with type 2 diabetes reported in Belgium (55). However, in the present study, we still used the criterion of a cutoff DN4 score ≥ 4, which has been validated for diagnosing DPNP in most countries (25).

Insulin was shown to possibly augment the mechanical responsiveness to mechanical stimuli and induce excitation in peripheral nerves in an *in vitro* study (56). In line with our findings, Huang et al. (40) reported that the use of insulin is an independent risk factor for DPN in patients with type 2 diabetes. On the other hand, metformin possibly attenuated neuropathic pain in a diabetic rat model (57). According to UK biobank data, the use of metformin had low odds of causing musculoskeletal pain in patients with type 2 diabetes (58). In the present study, the use of metformin was associated with a low pain score. Notably, long-term metformin use might cause vitamin B12 deficiency, and American Diabetes Association recommended monitoring vitamin B12 levels in patients with DPN (59).

Unlike prospective studies, we did not estimate case numbers before enrolling this retrospective cohort. Previous studies reported that DPNP prevalence was approximately 16.7% in type 2 diabetes (54, 55), and the cumulative mortality rate might be 11.5% in patients with DPNP and 8.5% in patients without DPNP during a median 3.1-year interval (19). The patient number of 2310 yielded statistical power > 0.85 for a median 6.2-year follow-up. However, CV diseases caused only 19.2% of total mortality in the present study, and the proportion of CV mortality was similar to the 19.6% in 2014 based on the Taiwan National Health Insurance Research Database (60). Despite the close relationship between neuropathic pain and CV risk, we only collected mortality data during follow-up, and could not assess CV event incidence. Based on the CV−total mortality ratio in the present study, the increased mortality risk in the patients with DPNP might not be driven only by CV diseases. Moreover, there are several limitations in the present study that should be acknowledged. First, we did not directly investigate the underlying mechanism between neuropathic pain and total mortality in patients with type 2 diabetes. Second, we did not collect all clinical information on DPNP, such as the duration of pain. Third, we did not investigate the effects of specific interventions for DPNP to improve prognosis in the observation study. Fourth, we aimed to use the convenient DPNP symptom scores to predict long-term mortality. A two-stage screening model was recommended for diagnosing DPN (6). However, we only collected questionnaire data but did not further diagnose DPN using electrodiagnostic tests. Finally, the ID pain and DN4 questionnaires are screening tools for diagnosing DPNP instead of estimating DPNP severity. Therefore, we could not speculate that a high score was correlated with high severity in DPNP.

## Conclusion

In the present study, we found that diagnosis of DPNP using the ID pain and DN4 were predictive of mortality. Furthermore, there was high consistency between the application of the ID pain and DN4 scores when assessing neuropathic pain among outpatients with type 2 diabetes. However, the discrepancy in the diagnosis of DPNP weakened the prediction of mortality. Screening for DPNP by the ID pain and DN4 questionnaires is helpful for categorizing the long-term risk of mortality in patients with type 2 diabetes. Further larger-scale studies are required to fully investigate the potential mechanisms between neuropathic pain and mortality.

## Data availability statement

The raw data supporting the conclusions of this article will be made available by the authors, without undue reservation.

## Ethics statement

The studies involving human participants were reviewed and approved by The Institutional Review Board of Taichung Veterans General Hospital. Written informed consent for participation was not required for this study in accordance with the national legislation and the institutional requirements.

## Author contributions

YL and IL conceived and designed the study. YL and IL organized all data. YL, SL, and IL, analyzed and visualized the results. YL and SL wrote the manuscript. IL reviewed and edited the manuscript. IL is the guarantors of this work and, as such, had full access to all the data in the study and take responsibility for the integrity of the data and the accuracy of the data analysis.

## Funding

This research was funded by the Taichung Veterans General Hospital, Taiwan (grant number TCVGH-1113501C), the National Health Research Institute (grant number NHRI-EX111-10927HT), and the Ministry of Science and Technology, Taiwan (grant number MOST 110-2314-B-075A-004 -MY3). The funders had no role in the decision to publish the results.

## Acknowledgments

We thank the Diabetes Care Center of Taichung Veterans General Hospital for their support. Statistical analysis was performed by the Biostatistics Task Force of Taichung Veterans General Hospital, Taichung, Taiwan.

## Conflict of interest

The authors declare that the research was conducted in the absence of any commercial or financial relationships that could be construed as a potential conflict of interest.

## Publisher’s note

All claims expressed in this article are solely those of the authors and do not necessarily represent those of their affiliated organizations, or those of the publisher, the editors and the reviewers. Any product that may be evaluated in this article, or claim that may be made by its manufacturer, is not guaranteed or endorsed by the publisher.
